# A new connection: management of disconnected segments 5 and 6 bile leak via the cystic duct remnant

**DOI:** 10.1016/j.vgie.2022.11.005

**Published:** 2022-12-16

**Authors:** Andrew Canakis, Adnan A. Alseidi, Shayan S. Irani

**Affiliations:** 1 Division of Gastroenterology and Hepatology, University of Maryland School of Medicine, Baltimore, Maryland; 2 Division of Surgical Oncology, University of California, San Francisco, California; 3 Division of Gastroenterology and Hepatology, Virginia Mason Medical Center, Seattle, Washington

**Keywords:** FCSEMS, fully covered self-expandable metal stent, IR, interventional radiology

## Abstract

Video 1 Management of disconnected segments 5 and 6 bile leaks.

Management of disconnected segments 5 and 6 bile leaks.

## Introduction

Iatrogenic adverse events following cholecystectomy can lead to bile leaks in up to 1.1% of cases.^1^ Management of disconnected bile leaks often requires multidisciplinary management, especially because they are often complex injuries associated with high morbidity and mortality.^2^ ERCP with sphincterotomy and stenting has emerged as a reliable treatment modality even in refractory or complex leaks with a resolution rate over 90%.^3^ Nonetheless, type C biliary segments 5 and 6 injuries are rare and account for 9.7% of leaks that make it to surgery.^4^ When surgery is not an option because of severe comorbidities, long-term percutaneous drain placement is typically the only option. We describe an alternative/endoscopic approach in this situation (Video 1, available online at www.giejournal.org).

## Case

**Figure 1:**
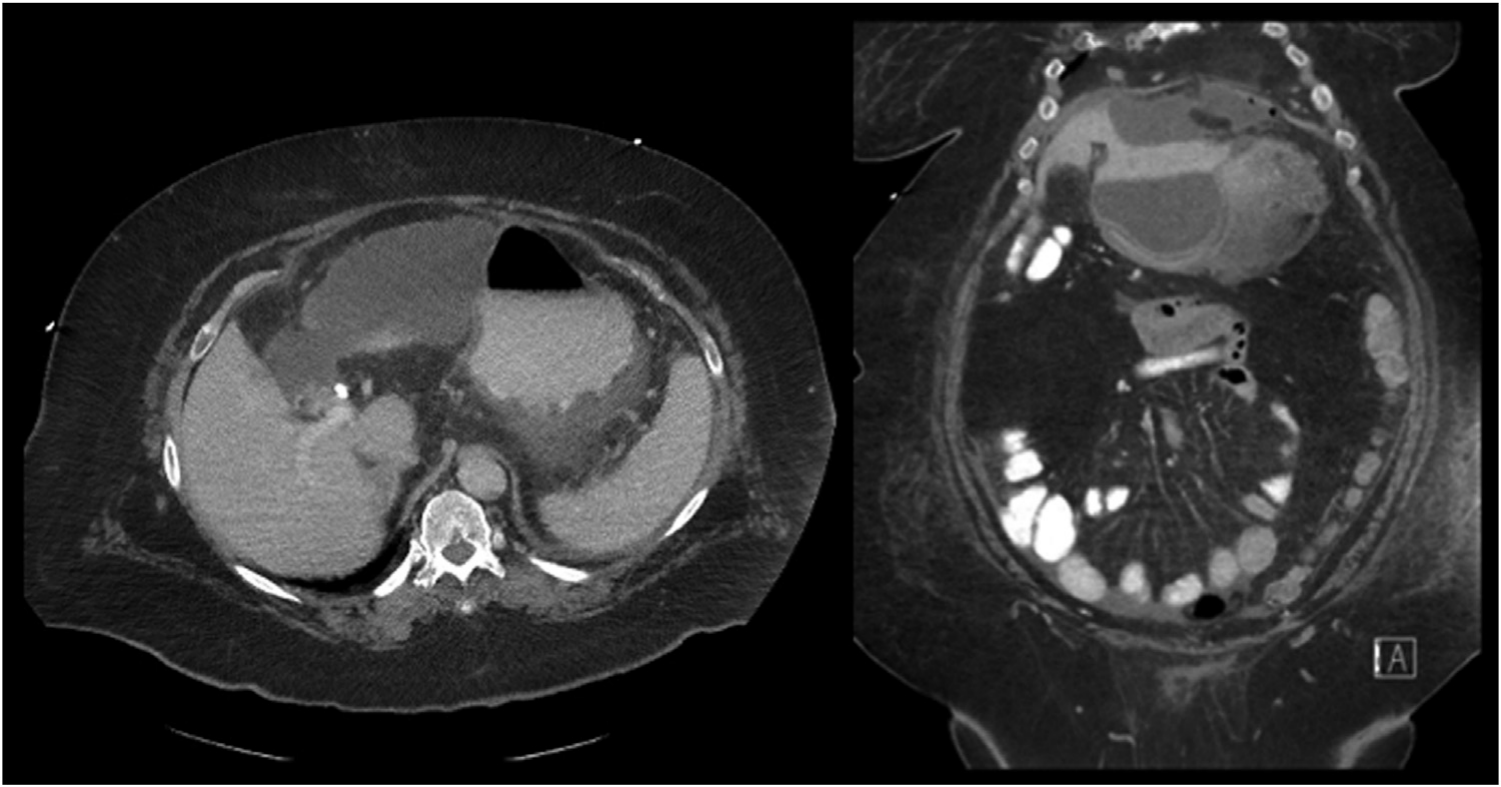
CT scan of abdomen showing the biloma.

**Figure 2:**
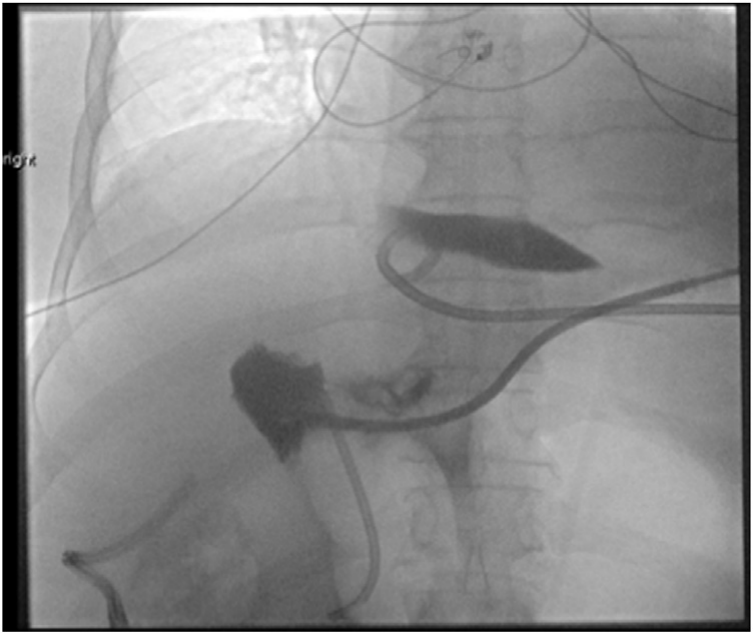
Interventional radiology–guided tube placement for biloma.

A 75-year-old woman with morbid obesity and multiple cardiac and pulmonary comorbidities was referred for endoscopic management of a disconnected segments 5 and 6 leak. She initially underwent a laparoscopic cholecystectomy for acute cholecystitis during which an intraoperative bile leak was noted. The source could not be identified, and a Jackson Pratt drain was placed. An ERCP at an outside facility demonstrated a cystic duct remnant leak. A 10F plastic stent was placed, but the leak did not resolve and 2 weeks later she presented with a large biloma requiring 2 interventional radiology (IR)–guided drains (Figs. 1 and 2). Over the next 2 months she continued to have persistent drain output (500-600 mL of bile per day) with 2 unsuccessful ERCPs, including placement of a fully covered self-expandable metal stent (FCSEMS).

**Figure 3:**
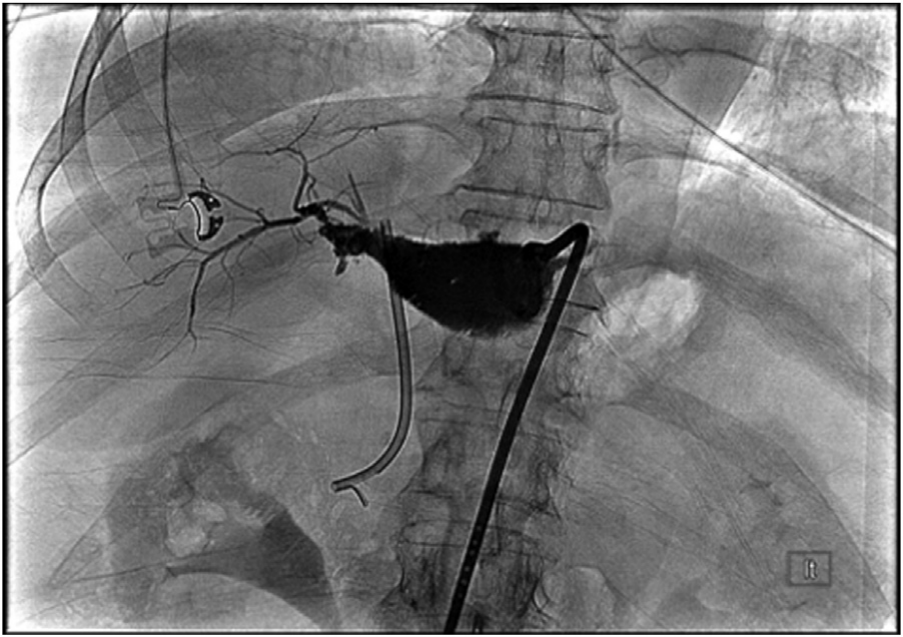
Tube check showing disconnected segments 5 and 6.

**Figure 4:**
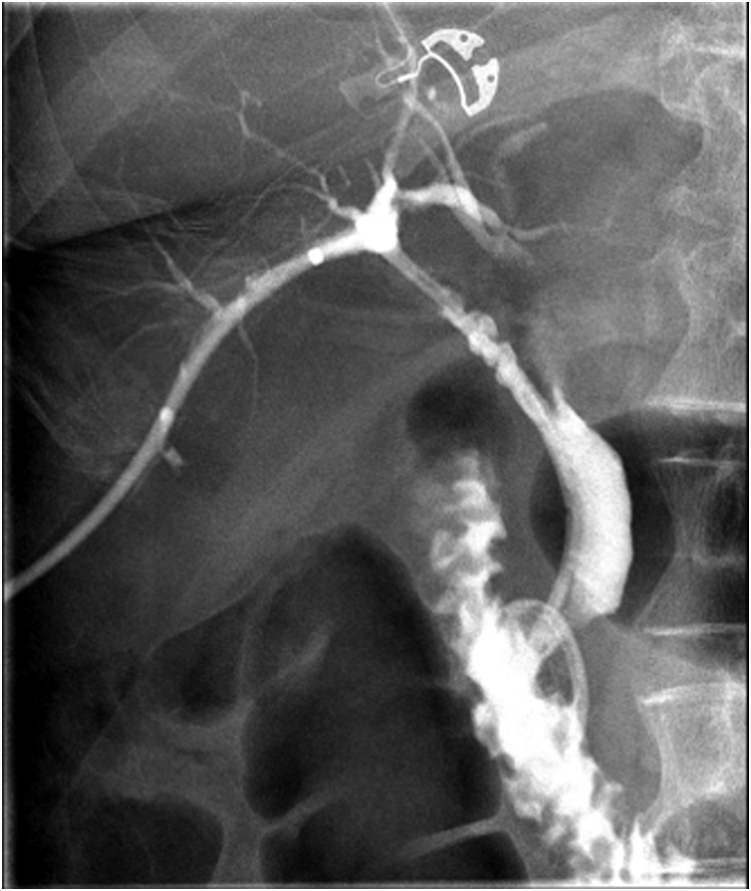
ERCP rendezvous allowing the placement of the percutaneous drain through the cyst duct into the duodenum.

In the setting of an ongoing bile leak, she was transferred for consideration of surgery. Evaluation of the IR drains revealed the problem; there was a leak from disconnected segments 5 and 6 to the gallbladder fossa (Strasberg type E and Stewary-Way class IV injury) that had been undetected at surgery and a concomitant cystic duct remnant leak that had confounded the picture (Fig. 3). An IR rendezvous ERCP was performed with placement of a percutaneous drain into the common bile duct via the cystic duct remnant. However, this failed to heal the segments 5 and 6 leak but did maintain cystic duct remnant patency (Fig. 4). Six months later she was still deemed a poor surgical candidate for a right partial hepatectomy because of her severe comorbidities. Consequently, her management options included long-term percutaneous drainage or endoscopic intervention. We proceeded with endoscopy given the dyscosmesis she experienced with the drain.

**Figure 5:**
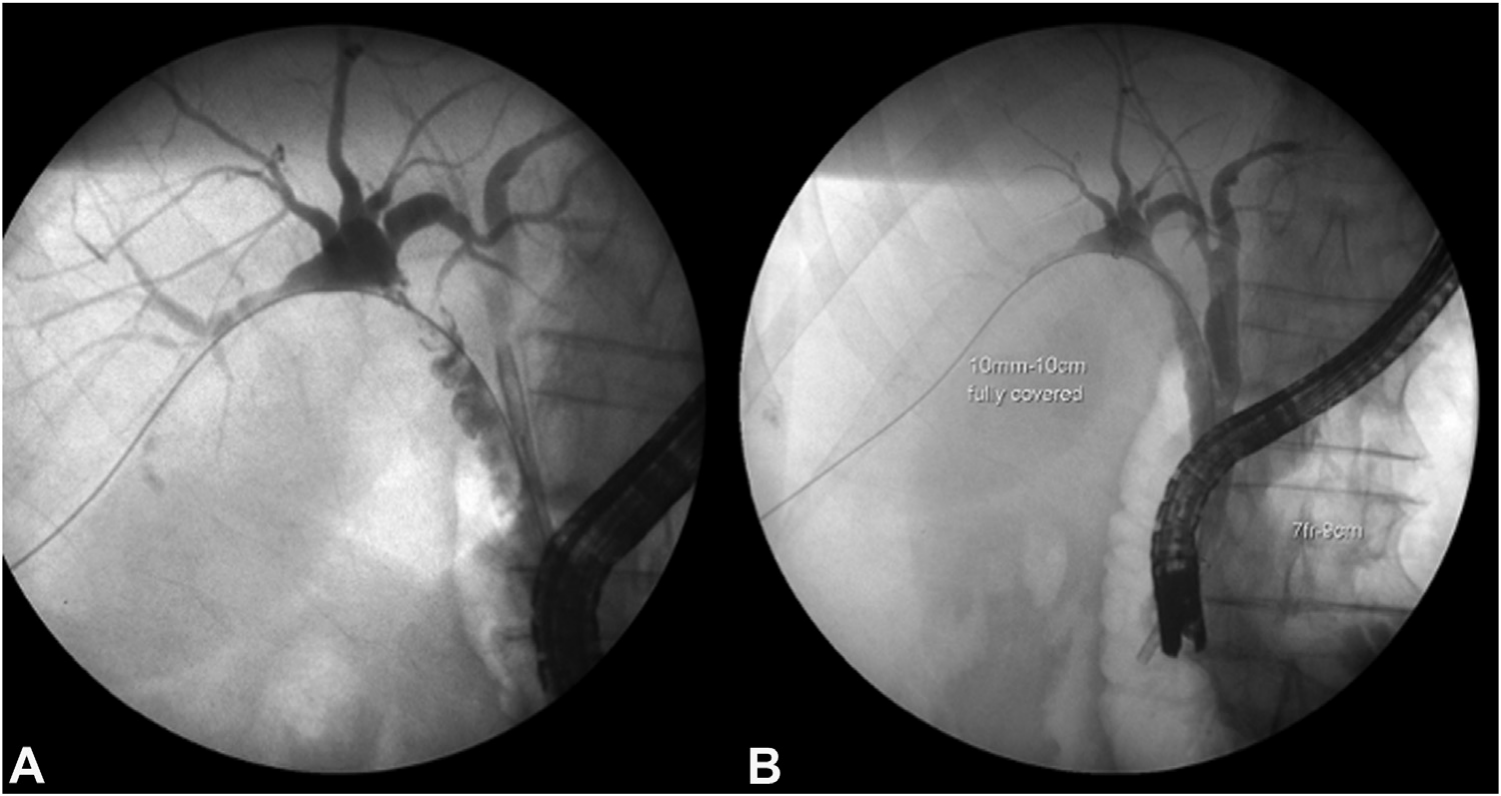
ERCP before **(A)** and after **(B)** placement of a biliary fully covered self-expandable metal stent.

At ERCP, we cannulated the cystic duct remnant to go through the gallbladder fossa to segment 5 and 6 into which a 10-mm × 10-cm biliary FCSEMS (Viabil; Gore, Flagstaff, Ariz, USA) was placed. However, it fell short of the ampulla, requiring an overlapping (Fig. 5) 10-mm × 4-cm FCSEMS to allow duodenal drainage. Two days later the bile leak resolved, and the percutaneous drain was removed the following week after an indwell of over 8 months.

Five months later, at the follow-up ERCP for stent exchange, the 10-mm × 10-cm FCSEMS was firmly adherent as it traversed the gallbladder fossa and could not be removed. To facilitate breaking up what was thought to be granulation tissue holding the stent in place, a 10-mm × 8-cm biliary FCSEMS with a different design (Wallflex; Boston Scientific, Natick, Mass, USA) was placed to help with stent-in-stent removal.

**Figure 6:**
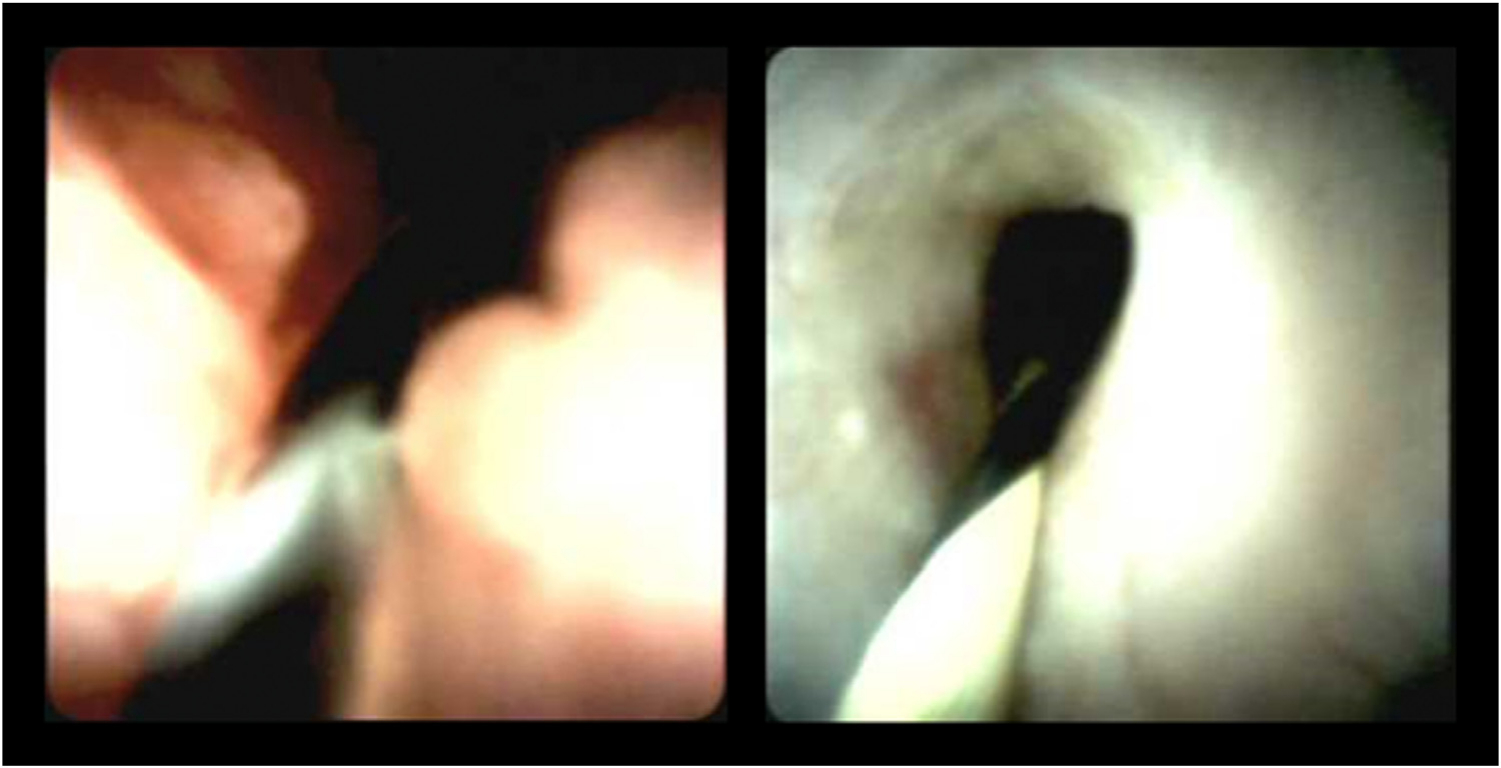
Cholangioscopy confirming epithelization of the tract.

Six weeks later both stents were successfully removed. Cholangioscopy at this time did not show adequate epithelialization of the newly created tract from the cystic duct remnant to segments 5 and 6 through the gallbladder fossa. Three 7F × 15-cm plastic stents were then placed for an additional 6 months and exchanged during 2 additional procedures (6 months apart). She was left stent-free after cholangioscopy confirmed epithelization (Fig. 6) of the tract and has remained asymptomatic without cholangitis and with normal liver function tests for the last 28 months.

## Conclusion

When surgery is not an option for the management of a complex bile leak to the gallbladder fossa from an aberrant segments 5 and 6 leak, it is possible to get a durable response with reconnecting this via the cystic duct remnant through the gallbladder fossa. In a multidisciplinary setting, with adequate surgical backup, endoscopic therapy can offer durable and less-morbid therapy options in the treatment algorithm of biliary duct transection. Further studies are required to see if this is a viable long-term option to surgery in certain patients.

## Disclosure

*Dr Irani is a consultant for Boston Scientific and Gore. All other authors disclosed no financial relationships.*

## Supplementary data

Video 1 Management of disconnected segments 5 and 6 bile leaks.
